# Mitochondrial superoxide: a key player in Alzheimer's disease

**DOI:** 10.18632/aging.100088

**Published:** 2009-09-14

**Authors:** Cynthia A. Massaad, Robia G. Pautler, Eric Klann

**Affiliations:** ^1^ Department of Molecular Physiology and Biophysics, Baylor College of Medicine, Houston, TX 77030, USA; ^2^ Center for Neural Science, New York University, NY 10003, USA

**Keywords:** Alzheimer's disease, Tg2576, SOD-2, Oxidative Stress, Mitochondria

Our recent study characterized a role for mitochondrial superoxide in the pathology of Alzheimer's disease [1]. Using the Tg2576 Alzheimer's disease (AD) mouse model in combination with a mouse that overexpresses the mitochondrial antioxidant enzyme superoxide dismutase (SOD-2), we showed that severe deficits in the spatial and associative memory of AD mice could be prevented by scavenging of superoxide. SOD-2 overexpression also resulted in a reduction in amyloid-β (Aβ) plaque deposition without affecting the levels of soluble and fibrillar Aβ. It did however lead to a reduction in the Aβ 42/40 ratio resulting in an Aβ pool composition less favorable for aggregation. These findings point towards the involvement of mitochondrial superoxide in AD pathology perhaps through its effects on Aβ processing. Here we discuss these findings and comment on the future directions that this research may lead to.

Alzheimer's disease (AD) is a progressive neurodegenerative disease characterized by the neuropathological deposition of extracellular amyloid-β (Aβ) plaques and intracellular tau neurofibrillary tangles [2]. The disease also is characterized by the devastating loss of memory and cognitive functions, which is thought to arise from the increased production of Aβ [3]. The neurotoxic role of Aβ has long been established [3], however, it is not clear how it contributes to the cognitive deficits characteristic of AD. In addition to Aβ, several studies have implicated oxidative stress in the etiology of AD.  Oxidative stress occurs mainly as  a result of overproduction of oxygen free radicals by mitochondria. The reactive oxygen species (ROS) superoxide is produced by complexes I and III of the mitochondrial respiratory chain [4], and is primarily detoxified by the mitochondrial antioxidant enzyme superoxide dismutase (SOD-2). Many lines of evidence support a pro-oxidant role for Aβ during AD [5,6]. Aβ promotes the production of ROS in several model systems by causing dysfunction of the mitochondrial respiratory chain. Despite overwhelming evidence for the pro-oxidant role of Aβ, several other studies demonstrate ROS involvement prior to amyloid pathology [7,8]. Oxidative stress has been shown to exacerbate multiple AD phenotypes. For example, partial SOD-2 deficiency has been shown to accelerate plaque deposition [9] and increase tau phosphorylation in AD mice models [10]. SOD-2 deficiency also has been shown to accelerate the onset of a number of behavioral deficits in hAPP mice [11].

In our recent study, we investigated the involvement of mitochondrial dysfunction in AD pathology by studying the offspring of Tg2576 AD model mice that were crossed with mice that overexpress SOD-2 [1]. We found that the elevated SOD-2 reduced age-dependent increases in hippocampal superoxide, presumably from mitochondria, in the Tg2576 mice [1]. The reduction of hippocampal superoxide in the Tg2576 mice by SOD-2 overexpression was correlated with the prevention of spatial and associative memory deficits, as measured by the Morris water maze and fear conditioning tests, respectively [1]. We also measured the levels of Aβ40 and Aβ42 in these mice. Although we observed no difference in the absolute levels of these two peptides in the Tg2576/SOD-2 mice compared to the Tg2576 mice, we found that the ratio of Aβ42 to Aβ40 was reduced by the overexpression of SOD-2, which is suggestive of an Aβ pool less favorable for aggregation [1]. This notion was supported by histological analysis demonstrating that overexpression of SOD-2 resulted reduced amyloid plaque deposition [1].

Our data are in agreement with several studies that demonstrate the involvement of mitochondrial ROS in AD [9-12]. These studies specifically link SOD-2 deficiency with increased AD pathology and reduced mitochondrial ROS with improved cognition [9-12]. Shortly following the submission of our studies for publication, two new supporting studies were published. The first one linked α-ketoglutarate dehydrogenase enzyme complex deficiency with increased mitochondrial ROS production and subsequent acceleration of the memory impairments and plaque deposition in Tg19959 mice [13]. The second study demonstrated mitochondrial ROS involvement in AD using a cross between the Tg19959 mouse model of AD and the SOD-2 overexpressing mice [14]. In agreement with our results, these authors demonstrated that memory impairments and increased plaque deposition in the Tg19959 mice were both alleviated by overexpression of SOD-2 [14]. These authors also showed that increased Aβ42 levels in the Tg19959 mice were resistant to overexpression of SOD-2 [14]. The authors interpreted this data as evidence that the facilitation of mitochondrial antioxidant responses results in resistance to Aβ and improvement of the global AD phenotype [14].

Our findings, together with the aforementioned studies, strongly suggest that mitochondrial ROS affect Aβ processing. One attractive possibility is the involvement of mitochondrial ROS in the assembly of fibrillar Aβ into plaques. This notion is supported by us and others, showing that quenching ROS does not affect fibrillar Aβ levels but reduces plaque deposition.

The involvement of oxidative stress in AD has long been known and investigated [5,6,15,16]. As a result of multiple studies linking AD to oxidative damage, the use of antioxidant therapy for AD has received considerable attention over the years. Clinical trials, however, have produced conflicting results concerning the therapeutic efficacy of antioxidant treatment for AD [17,18]. This may be due to either poor understanding of the pharmacokinetics or suboptimal specificity of the antioxidants compared with targeted pharmacological therapy. We and others have shown a specific involvement of mitochondrial superoxide in the etiology of AD [1,9-11,14]. Therefore designing antioxidants targeted towards mitochondrial ROS offers an attractive therapeutic approach for AD. An important consideration to keep in mind is that the SOD-2 overexpressing mice likely have reduced levels of mitochondrial ROS beginning at birth and hence, studying the offspring generated when these mice are mated with the Tg2576 AD mice shows that quenching mitochondrial superoxide is a preventive approach to the occurrence of AD. A more clinically relevant approach would be to determine the specific temporal window in which removal of mitochondrial ROS is most effective.

One could use either genetic or pharmacological approaches to determine whether removal of mitochondrial ROS reverses rather than prevents cognitive deficits in AD model mice. From a genetic standpoint, an attractive approach would be to study the progeny of Tg2576 AD mice crossed to a mouse that could be induced to express SOD-2 on demand (for example a mouse that overexpresses SOD-2 in response to tetracycline treatment - tet-on-SOD-2). Studying AD model mice that could overexpress SOD-2 at any age would permit one to determine whether diminishing mitochondrial superoxide after the onset of AD phenotypes could reverse symptoms of AD, such as cognitive dysfunction and plaque deposition.

From a pharmacological standpoint, the design of antioxidants specifically targeted to mitochondria to quench mitochondrial ROS is necessary. Pharmacological catalytic scavengers of superoxide and hydrogen peroxide have proven quite effective in reversing age-related cognitive deficits [19]. In the context of AD, these compounds only have been tested in organotypic hippocampal cultures treated with Aβ42 or Aβ40 [20]. In support of our findings, SOD/catalase mimetics were shown to be effective against Aβ-induced neurotoxicity [20]. These mimetics, however, are not specific to mitochondrial ROS and do not provide information for determining the optimal temporal window *in vivo* for treatment of AD. A set of synthetic plastoquinone derivatives recently were shown to specifically target the mitochondria and act as potent antioxidants at low concentrations [21,22]. The antioxidant properties of these derivatives (termed SKQs) have been investigated in the context of senescence and age-related impairments such as blindness [23], ischemia [24], stroke [24] and tumor development [25]. Although these agents can act as potent pro-oxidants at higher concentrations and optimization of the antioxidant therapeutic window is necessary [21,22], they appear to be promising in terms of decelerating senescence and treating age-related diseases [21-27]. In the context of our data, which show an involvement of mitochondrial ROS in AD pathology, the use of the SKQ agents for the treatment of AD should be investigated.

In conclusion, our findings have reinforced the idea that mitochondrial superoxide plays a critical role in the pathological events following Aβ elevation during AD. More specifically, we were able to show that increasing the expression of the mitochondrial antioxidant enzyme SOD-2 prevents memory deficits and amyloid plaque deposition associated with AD. Moreover, our findings suggest that mitochondrial superoxide may be involved in Aβ processing, perhaps at the level of accumulation of fibrillar Aβ into plaques. The next challenge will be to determine whether quenching mitochondrial superoxide, in addition to being preventative, can be therapeutic for the treatment and reversal of cognitive dysfunction in AD.
